# Methylation of *Cdkn1c* may be involved in the regulation of tooth development through cell cycle inhibition

**DOI:** 10.1007/s10735-018-9785-0

**Published:** 2018-07-16

**Authors:** Qiulan Li, Yue Guo, Mianfeng Yao, Jun Li, Yingyi Chen, Qiong Liu, Yun Chen, Yuanyuan Zeng, Bin Ji, Yunzhi Feng

**Affiliations:** 1 Department of Stomatology, Second Xiangya Hospital, Renmin Middle Road, Changsha, 410011 Hunan China; 2 Department of Oral Medicine, Xiangya Hospital Central South University, Changsha, 410083 Hunan China

**Keywords:** *Cdkn1c*, Tooth development, DNA methylation, Cell cycle, Cell differentiation

## Abstract

*Cdkn1c*, a member of the *Cip*/*Kip* cyclin-dependent kinase inhibitor family, is critically involved in regulating cell cycle and cellular differentiation during development in mammals. However, the functional role of *Cdkn1c* and the underlying mechanisms by which *Cdkn1c* affects odontogenesis remain largely unknown. In our study, we found that *Cdkn1c* expression dynamically changes from embryonic day 11.5 (E11.5) to postnatal day 3 (P3), and exhibits tissue-specific expression profiles. Evaluation of CDKN1C protein by immunohistochemistry and western blot, revealed that CDKN1C protein expression peaks at P3 and then is reduced at P5 and P7. Interestingly, we observed that CDKN1C expression is higher in immature odontoblasts than preodontoblasts, is lower in mature odontoblasts, and is practically absent from ameloblasts. We evaluated cell cycle progression to further investigate the mechanisms underlying CDKN1C-mediated regulation of odontogenesis, and found that pRB, cyclin D1 and CDK2 expression decreased from P1 to P3, and reduced at P5 and P7. In addition, we observed increased methylation of *KvDMR1* at P1 and P3, and reduced *KvDMR1* methylation at P5 and P7. However, the methylation levels of *Cdkn1c*-sDMR were relatively low from P1 to P7. In summary, we demonstrated that *Cdkn1c* expression and methylation status may be involved in early postnatal tooth development through regulating the cell cycle inhibition activity of *Cdkn1c*. Notably, *Cdkn1c* expression and methylation may associate with cell cycle exit and differentiation of odontoblasts.

## Introduction

Tooth development involves a continuous and reciprocal series of interactions between dental epithelium and mesenchyme (Soukup et al. 2008; Lee et al. 2016); this cycle depends on the tightly regulated spatiotemporal expression of specific genes (Zhang et al. 2009; Zhou et al. 2017; Du et al. 2016). Cell-cycle-associated genes, including genes of the *Cip*/*Kip* family of cyclin-dependent kinase inhibitors, are critical mediators and regulators of odontogenesis (Kumamoto et al. 2001). *Cip*/*Kip* family genes are known to be important in differentiation of odontoblasts and ameloblasts (Lee et al. 2009; Iwamoto et al. 2017) and facilitate dental mineralization (Yin et al. 2014). However, little is known about the role of *Cdkn1c*, a recently discovered *Cip*/*Kip* family member, in odontogenesis.

p57^KIP2^, encoded by the *Cdkn1c* gene, is a major regulator of cell cycle progression (Hildebrand et al. 2012), and is vital in cell proliferation and differentiation (Pateras et al. 2009). p57^KIP2^ inhibits cyclin/cyclin-dependent kinase (CDK) complexes in the G1 phase, leading to the inhibition of phosphorylation of the retinoblastoma (Rb) protein, and resulting in suppression of E2F family members in the mid-G1 phase (Tury et al. 2012). In addition to its effect on growth arrest, p57 plays distinct functions in the development of several organs (Chung and Park 2016; Mademtzoglou et al. 2017; Stantzou et al. 2017), especially hair follicles (Purba et al. 2017), which share a similar formation process as teeth. Mice deficient for *Cdkn1c* expression display hyperplasia in several organs, and are not viable (Yan et al. 1997). In humans, abnormal expression of the *CDKN1C* gene is primarily associated with growth disorder syndromes, including Beckwith–Wiedemann syndrome (Bastaki et al. 2016) and Silver–Russell syndrome (Nakashima et al. 2015). Notably, these diseases are always accompanied by dental abnormalities, such as enamel defects, delayed dental age, and diminished mandibular development (Abeleira et al. 2011; Bergman et al. 2003).

In addition to genetic factors, epigenetics may also affect odontogenesis, as suggested by differences in tooth morphology seen in monozygotic twins (Townsend et al. 2009; Hughes et al. 2014). DNA methylation of gene loci is an epigenetic modification (Deaton and Bird 2011) that has profound and widespread effects on cellular differentiation, genomic imprinting, and tissue-specific gene expression (Suelves et al. 2016; Wu et al. 2016). Su et al. (2016) reported that DNA methylation participates in odontogenesis; they identified 2469 genes that are differentially methylated during pig tooth development from embryonic day 50–60 (E50–E60). Treatment of human dental pulp cells with 5-Aza-2′-deoxycytidine, an inhibitor of DNA methyltransferase activity, decreased cell proliferation and enhanced odontogenic differentiation (Zhang et al. 2015). Moreover, Liu et al. reported that DNA methylation facilitates the osteogenic differentiation of human periodontal ligament stem cells (Liu et al. 2016). The expression of *Cdkn1c* is also regulated by methylation, and is mainly associated with two differentially methylated regions (DMR) in mice: *Cdkn1c*-sDMR and *KvDMR1* (Beatty et al. 2006; Bhogal et al. 2004). Therefore, investigating the methylation levels of *Cdkn1c* during odontogenesis may elucidate mechanisms of tooth development.

*Cdkn1c* gene is a maternally expressed imprinted gene (Matsuoka et al. 1996) and is essential for normal development (Bhogal et al. 2004). The spatiotemporal expression of the imprinted genes *Dlk1* and *Igf2* coincide with their methylation levels during tooth development (Khan et al. 2012). This suggests that imprinted genes and their methylation levels may be closely involved in odontogenesis. However, the expression and methylation status of *Cdkn1c* during odontogenesis remain virtually unexplored.

In this study, we characterize the expression of *Cdkn1c* gene during the early postnatal stages of the mouse first mandibular molar, and we investigate the underlying mechanisms connecting *Cdkn1c* expression with tooth development.

## Materials and methods

### Gene expression omnibus (GEO) data acquisition and processing

*Cdkn1c* gene expression data during tooth development was obtained from datasets available in the NCBI GEO database: GSE32321 (O’Connell et al. 2012), GDS4453 (Lachke et al. 2012), GSE76316 (Pantalacci et al. 2017) and GSE19488 (Sasaki et al. 2010) (http://www.ncbi.nlm.nih.gov/geo/). *Cdkn1c* mRNA expression z-Score was used for statistical analysis of differential gene expression (Sun et al. 2014).

### Animals

Young postnatal (P1, P3, P5, and P7) BALB/c mice were obtained from Hunan SJA Laboratory Animal Co. The Animal Ethics Committee of the Central South University provided the approval for all experimental procedures. Animals were humanely euthanized by cervical dislocation. Mandibles were isolated and immediately fixed overnight in 4% paraformaldehyde (Biosharp, China) buffered with 0.1 M PBS (Zhongshan Goldenbridge Biotechnology, China), pH 7.4, at 4 °C. The first mandibular molar tooth germ was dissected using a stereomicroscope.

### Histological and immunohistochemical analysis

After fixation for 24 h, the mandibles were decalcified in 10%, pH 7.4 EDTA for 5–7 days. Mandibles were placed into in a graded ethanol series to dehydrate, and were then embedded in paraffin. Finally, serial sagittal sections were obtained at 4 µm thickness; standard hematoxylin and eosin staining (HE) was employed to examine tissue morphology.

The process of immunohistochemical staining was previously described (Guo et al. 2016). In brief, antigen retrieval was performed by deparaffinizing the sections and incubating the sections with sodium citrate buffer (pH 6.0). Sections were blocked with 3% hydrogen peroxide and subsequently with 5% (v/v) bovine serum albumin (BSA) for 30 min, respectively. Sections were then incubated overnight at 4 °C with a monoclonal antibody to p57^KIP2^ (#ab75974, 1:1000, Abcam, USA). Sections were then incubated sequentially with agent 1 and agent 2 (#PV-9000-D, Zhongshan Goldenbridge Biotechnology, China). Finally, sections were visualized using a DAB staining kit (#ZLI-9018, Zhongshan Goldenbridge Biotechnology, China). Photomicrographs were obtained for further analysis. PBS was used as a negative control.

### Western blot analysis

We isolated 4–20 first mandibular molar tooth germs from each postnatal stage (P1, P3, P5, P7). Total protein was isolated as previously described (Guo et al. 2017; Liu et al. 2018). In brief, total protein lysates were obtained using a lysis buffer composed of 180 µl of RIPA Buffer (Thermo Scientific, USA), 20 µl protease inhibitor cocktail (Bimake, USA), and 2 µl phosphatase inhibitor cocktail (Bimake, USA). Protein concentration was detected using the BCA method. Total protein lysates from each postnatal stage were resolved by SDS-polyacrylamide gel electrophoresis on an 8% w/v gel (Amresco, USA), and were then transferred onto PVDF membranes (Millipore, USA). After transfer, membranes were blocked in 5% fresh skim milk (Yili, China) and incubated overnight at 4 °C in TBST + 5% BSA supplemented with primary antibodies against p57^KIP2^ (#ab75974, 1:1000, Abcam, USA), Rb (#ab181616, 1:2000, Abcam, USA), Phospho-Rb (Ser780) (#D59B7, 1:1000, Cell Signaling Technology, USA), CDK2 (#78B2, 1:1000, Cell Signaling Technology, USA), Cyclin D1 (#92G2, 1:1000, Cell Signaling Technology, USA), or α-tubulin (#sc-23948, 1: 2000, Santa Cruz, USA). Membranes were then incubated for 1 h at 37 °C with an anti-rabbit peroxidase-conjugated secondary. Protein bands were visualized using an Immobilon western chemiluminescent HRP substrate (Millipore, USA).

### Quantification of global DNA methylation

Genomic DNA was extracted from tooth germ (n = 4–10 from each stage) using a DNeasy Blood and Tissue Kit (#69504, Qiagen, Germany). Global methylation levels were evaluated using a MethylFlash™ Methylated DNA Quantification Kit (Colorimetric) (#P-1034, Epigentek, USA). Unmethylated (negative) control DNA (20 ng), a graded series of methylated (positive) control DNA, and purified sample DNA (about 100 ng) were incubated with a bonding solution in strip wells in duplicate. The methylated fraction of DNA was measured with dilute 5-mC capture and detection antibodies. A color developing solution was added and the absorbance at 450 nm was measured using a microplate spectrophotometer. The average of the duplicate measures was used for analyses. A standard curve was using the positive control series and optimal slope was calculated. The amount of methylated DNA was calculated according to the formula provided by the kit manufacturer.

### Bisulfite sequencing PCR

Bisulfite conversion of DNA was carried out with the EpiTect Fast DNA Bisulfite Kit (Qiagen, Germany). The methylation status of two differentially methylated regions (*Cdkn1c*-sDMR and *KvDMR1*) associated with *Cdkn1c* imprinting in mice was analyzed in this study. These methylated regions were amplified using the following specific primer pairs, as previously reported (Van de Pette et al. 2017): sDMR-F: 5′-GATTAGTATAATGTAGTATTTTTAGTTT-3′; sDMR-R: 5′-AACTATACCCAACTCCATAATC-3′; *KvDMR1*-F: 5′-TTAAGGTGAGTGGTTTAGGATA-3′; *KvDMR1*-R: 5′-AAACCACTATAAACCCAC ACA-3′. PCR products were separated by electrophoresis on a 1.5% agarose gel; bands were recovered with a gel extraction kit (Sangon, China). Extracted fragments were cloned it into T-vectors (Tiangen, China), and transformed into competent bacteria (DH-5a *Escherichia coli*). DNA from single bacterial colonies was amplified by PCR using vector-specific primers; DNA methylation was analyzed by sequencing the PCR products.

### Statistical analysis

All statistical analyses were performed using SPSS 20.0 software (IBM SPSS; Armonk, NY, USA). Data are expressed as means ± standard deviation (SD). Significant differences between two groups were analyzed using an independent t-test; multiple comparisons were performed using the least-significant difference (LSD) test. A p-value of *P* < 0.05 indicated a statistically significant result.

## Results

### *Cdkn1c* expression changes dynamically during molar tooth development

**Fig. 1:**
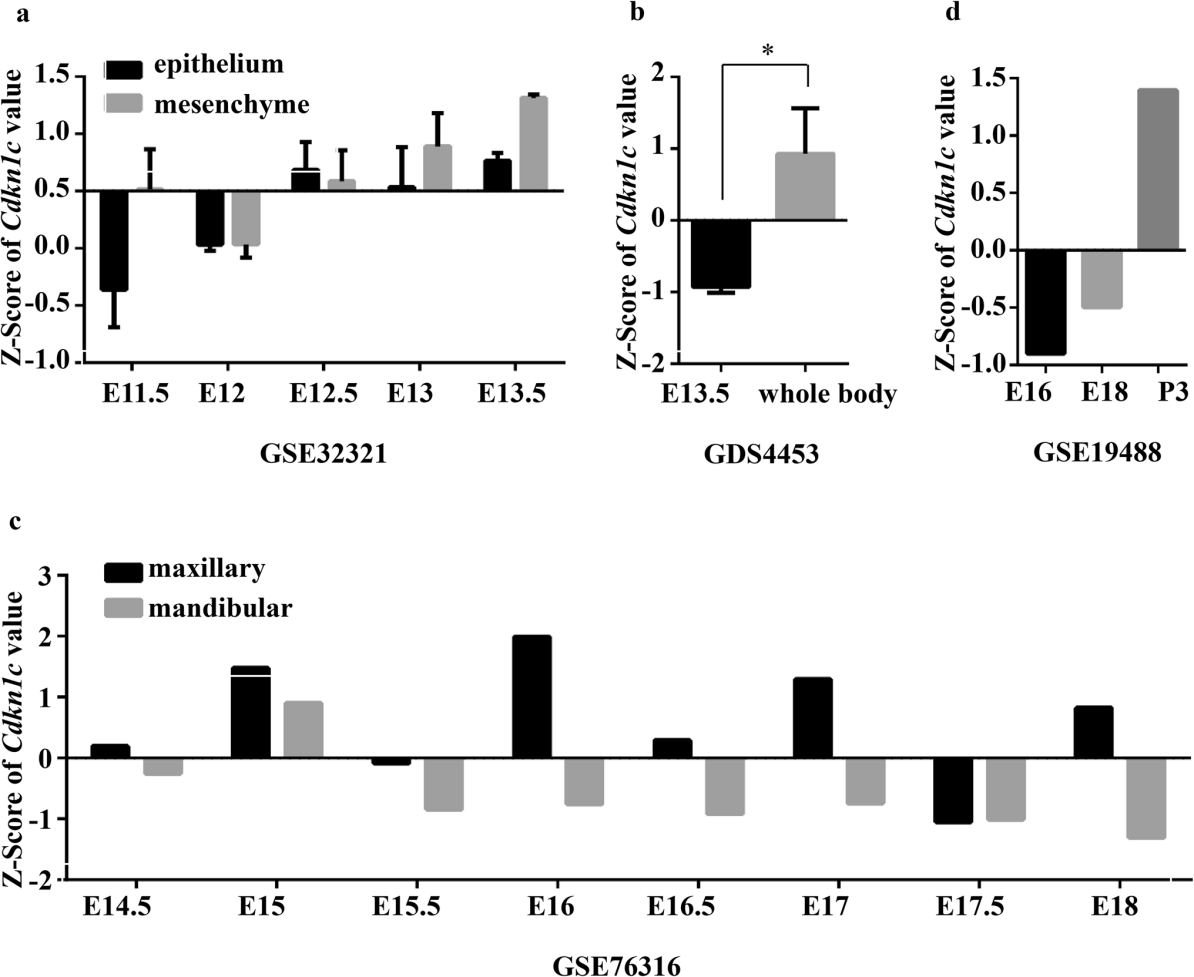
Analysis of *Cdkn1c* expression in tooth germ from the gene expression omnibus (GEO) database. **a** Expression levels of *Cdkn1c* in dental epithelial and mesenchymal tissue at embryonic day 11.5–13.5 from C57BL/6 mice (GSE32321). **b** Expression levels of *Cdkn1c* in tooth germ tissue from ICR mice at embryonic day 13.5 and matched whole embryo body without tooth germ (GDS4453). **c** Expression levels of *Cdkn1c* in CD1 mouse lower and upper first molar for eight consecutive stages (embryonic day 14.5–18) (GSE76316). **d** Expression levels of *Cdkn1c* in ICR mice at pre- and post-natal stages (embryonic day 16 to postnatal day 3) from dental papillae of mandibular first molar tooth germs (GSE19488). *E* embryonic day, *P* postnatal day, *P < 0.05

To investigate whether the *Cdkn1c* gene is involved in tooth development, we analyzed four GEO datasets: GSE32321 (O’Connell et al. 2012), GDS4453 (Lachke et al. 2012), GSE76316 (Pantalacci et al. 2017) and GSE19488 (Sasaki et al. 2010) (Fig. 1). Mouse tooth formation starts morphologically at embryonic day 11.5 (E11.5), and involves an interaction between dental epithelium and mesenchyme at the site of the future tooth; the region progresses through the bud stage, cap stage, bell stage, and secretory stage (O’Connell et al. 2012). The expression levels of *Cdkn1c* in dental epithelial and mesenchymal tissue began to increase at E11.5–E13.5, and were found to differ from each other at specific stages (Fig. 1a). However, *Cdkn1c* expression was still significantly lower than that of the whole embryo body without tooth germ at E13.5 (Fig. 1b). To further explore the existence of spatiotemporal expression of *Cdkn1c* in tooth development, we found the expression levels of molar *Cdkn1c* constantly changed during E14–E18, and were different between the upper and lower molars (Fig. 1c). After birth, *Cdkn1c* expression increased (Fig. 1d). These data indicate that *Cdkn1c* gene may be involved in odontogenesis.

### Involvement of p57 in early postnatal tooth development

**Fig. 2:**
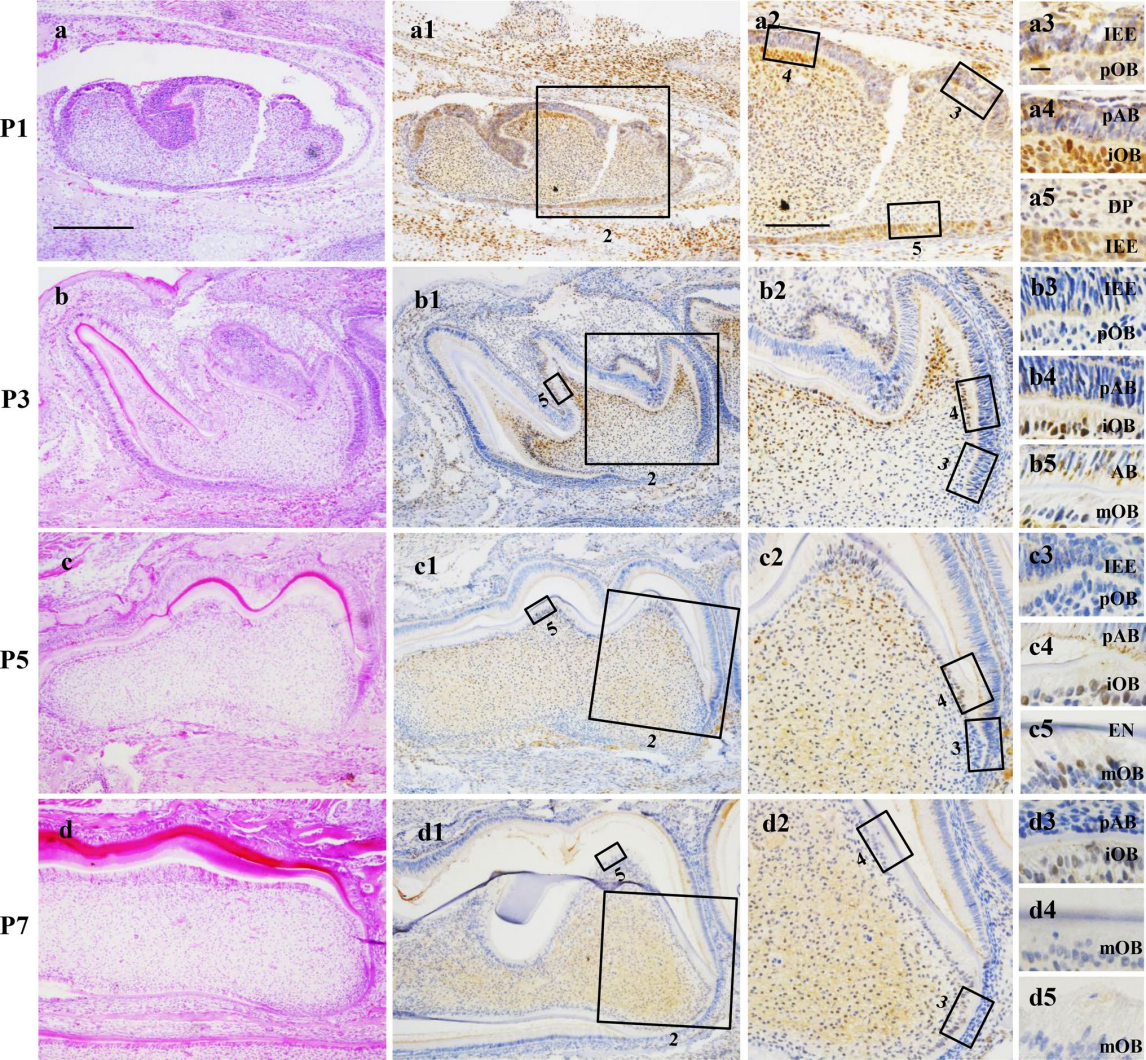
Developmental localization of p57 during mouse molar development. Immunohistochemistry for p57 in the lower first molar at postnatal day 1–7 (P1–P7). **a**–**d** HE staining, **a1**–**d5** Immunohistochemistry staining. **a2**–**d2** higher magnified views of *boxed areas* indicated as 2 in **a1**–**d1**; the column at the right side is higher magnified views of boxed areas indicated as 2, 3, and 4 in **a1**–**d1** and **a2**–**d2**, respectively. The tooth germ was in the late bell stage at P1 and P3, and entered the secretory stage at P5 and P7. p57 protein is intensely expressed in odontoblasts, although other pulpal cells also show weak positive reactions **a1**–**d1**. There was almost no positive p57 expression in ameloblasts **a2**–**d2** and preodontoblasts **a3**–**c3**. p57 expression was highest in immature odontoblasts **a4**–**c4, d3**, and was lowest in mature odontoblasts **b5, c5, d4**; p57 expression may have already been down-regulated in the mature odontoblasts of the pulp horn at P7 when primary dentin formation is almost completed **d5**. Note the positive expression in inner enamel epithelium at P1 **a5**. *IEE* inner enamel epithelium, *DP* dental pulp, *AB* ameloblast, *pAB* preameloblast, *pOB* preodontoblast, *iOB* immature odontoblast, *mOB* mature odontoblast, *EN* enamel, *P* postnatal day. Scale bars = 200 µm in **a** for **a–d, a1–d1**, 80 µm in **a2** for **a2–d2**, 10 µm in **a3** for **a3–d5**

From the HE staining (Fig. 2a–d) we can know that the tooth germ was in the late bell stage at P1 and P3, and paved the way for the morphogenesis of the entire dental structure. At P5 and P7, the first molar entered the secretory stage, and began to produce a large amount of hard tooth tissue. The p57 expression was highly expressed at P1 and P3, and was reduced at P5 and P7 (Fig. 2a1–d1). Almost no positive staining for p57 was observed in ameloblasts from P1 to P7, except for some undifferentiated inner enamel epithelial cells at P1 (Fig. 2a5). p57 was primarily expressed in odontoblasts. Odontoblasts can be classified in three subtypes (Quispe-Salcedo et al. 2012): preodontoblasts, immature odontoblasts, and mature odontoblasts. p57 was intensely expressed in immature odontoblasts (Fig. 2a4–c4, d3), although other pulpal cells also showed weak positive staining. In contrast, p57 expression was greatly reduced in mature odontoblasts (Fig. 2b5, c5, d4), and was almost absent from the mature odontoblasts of the pulp horn at P7 (Fig. 2d5). However, p57 was nearly negative in preodontoblasts (Fig. 2a3–c3). These data suggest that p57 function might be critically required during the early phases of odontoblast differentiation.

### p57 participates in tooth development via its activity as a cell cycle inhibitor

**Fig. 3:**
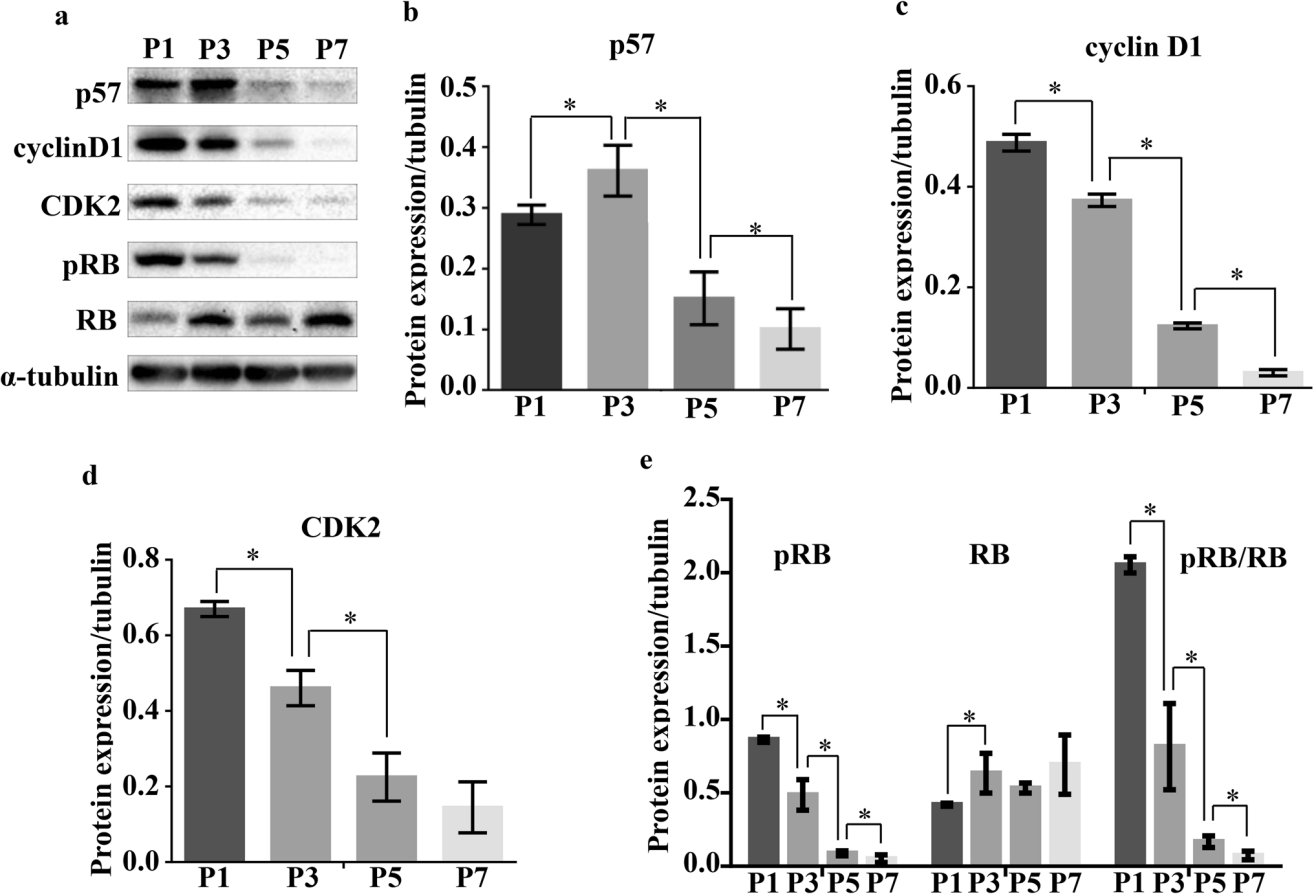
Expression of downstream signaling pathways related to cell cycle and p57 during mouse molar development. **a** Expression levels and relative abundance of p57, cyclin D1, CDK2, RB, and pRB in the mandibular first molar germ at postnatal day 1–7. **b**–**e** From postnatal day 1–3, the expression of p57 was increased, while expression of cyclin D1, CDK2, and pRB are decreased. Expression of p57, cyclin D1, CDK2, RB, and pRB was decreased at postnatal day 5 and 7. α-tubulin was used as a control. Data were pooled from three independent experiments with error bars designating standard deviation of the mean. *P* postnatal day, *P < 0.05

To explore the mechanisms underlying the involvement of p57 in tooth development, we performed western blot analysis to evaluate the activation of signaling pathways related to the cell cycle at P1, P3, P5, and P7 (Fig. 3). The expression of p57 was consistent with the results from the IHC staining, increasing from P1 to P3, peaking at P3, then sharply falling at P5 and P7 (Fig. 3a). In a similar fashion to p57, expression levels of cyclin D1, CDK2, and pRB were the lowest at P5 and P7. However, expression patterns of cyclin D1, CDK2, and pRB decreased from P1 to P3 (Fig. 3b–d), suggesting that p57 negatively regulates the expression of CDK2, cyclin D1, and RB phosphorylation from P1 to P3, and is no longer effective from P5 to P7.

### *Cdkn1c* methylation regulate *Cdkn1c* expression during mouse tooth development

**Fig. 4:**
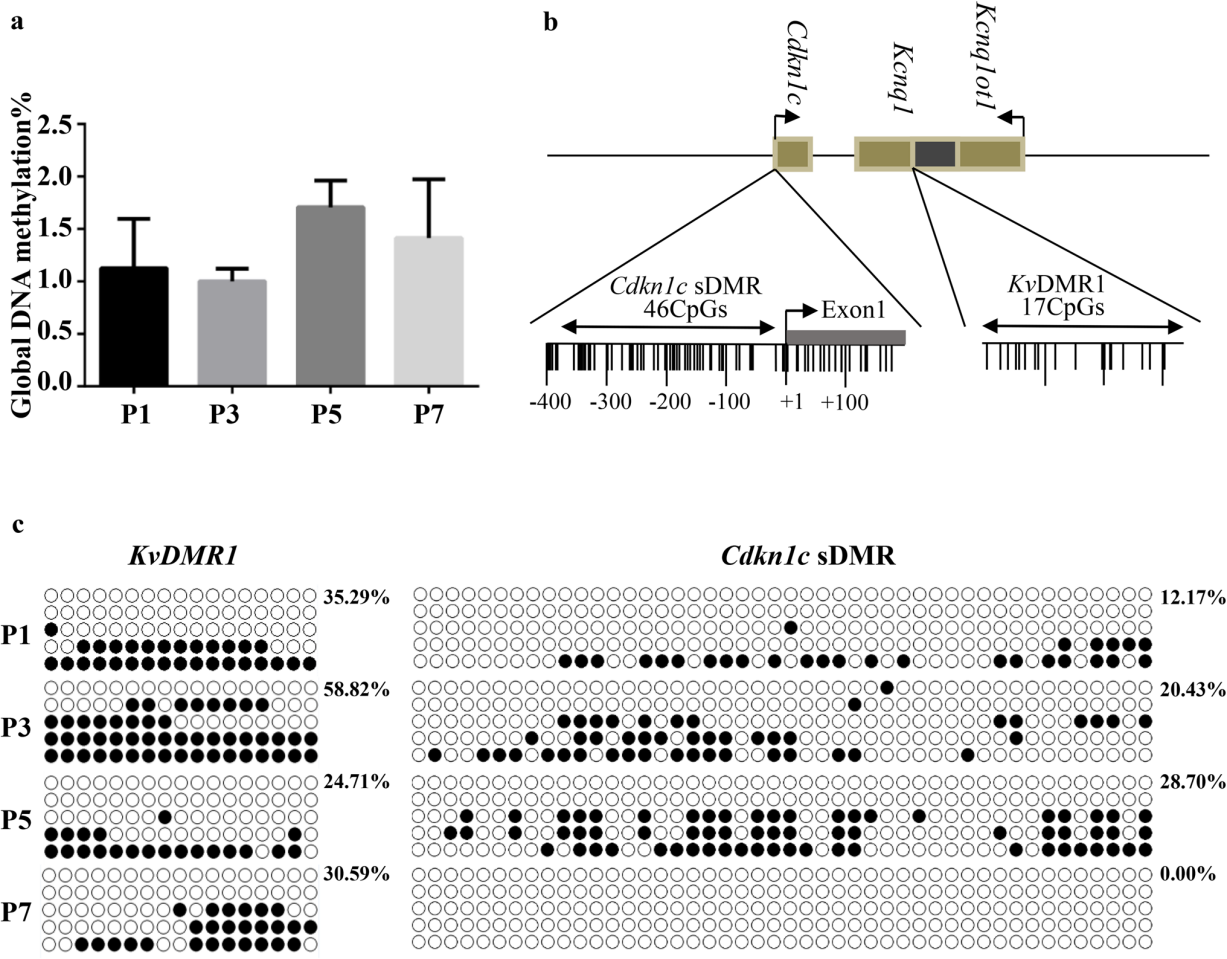
Global and *Cdkn1c* specific DNA methylation analysis during mouse tooth development. **a** Quantification of 5-mC in genomic DNA isolated from postnatal day 1–7 mouse first molar tooth germ. The data are average values ± standard deviation (SD) from three different assays. The LSD test revealed no statistically significant differences between the two groups. **b** Schematic diagram of two differentially methylated regions (DMRs) which associate with *Cdkn1c* imprinting in the mouse. The horizontal bars indicate the positions of the two DMR; the narrow vertical bars represent the position of CpG dinucleotides. For *Cdkn1c* sDMR, we analyzed 361 bp containing 46 CpGs; the *KvDMR1* region contains 341 bp and 17 CpGs. **c** Bisulfite sequence analysis showing the methylation status of the two DMRs that regulate *Cdkn1c* imprinted expression (*KvDMR1* and *Cdkn1c* sDMR). Each circle indicates an individual CpG dinucleotide. Each row of circles corresponds to an individual clone of the bisulfite-PCR product. Empty and filled circles indicate unmethylated and methylated CpGs, respectively. The numbers beside the bisulfite-sequencing profiles indicate the percentage of methylated CpG sites. P: postnatal day

To analyze whether the DNA methylation is associated with tooth development, we evaluated global DNA methylation levels in mouse first molar germ at P1, P3, P5, and P7. The overall levels of DNA methylation during tooth development were low (Fig. 4a), but there was still a dynamic change at each stage, although the average DNA methylation level showed no significant differences between each stage. This suggests that DNA methylation may be involved in odontogenesis.

Since global DNA methylation level does not reflect the methylation level of a specific gene, we further examined the methylation level of two differentially methylated regions (DMRs) of the *Cdkn1c* gene (Fig. 4b, c): *Cdkn1c*-sDMR and *KvDMR1*. The *KvDMR1* region contains 17 CpGs within a 341-bp sequence. *KvDMR1* showed dense methylation at P3, but sparse methylation on P5 and P7; this was consistent with the p57 expression levels on those days. Maternal methylation of *KvDMR1* suppresses the expression of *Kcnq1ot1* and subsequently allows for the expression of *Cdkn1c* (Fitzpatrick et al. 2002). Together, the methylation levels of *KvDMR1* region may play a role in regulating the expression of the *Cdkn1c* gene during odontogenesis. The *Cdkn1c* sDMR region is a 361-bp region containing 46 CpGs. The methylation levels of the *Cdkn1c* sDMR region were relatively low in P1, P3, and P5, and the region was un-methylated in P7. Since the methylation levels at *Cdkn1c* sDMR are not a precondition for the imprinted expression of *Cdkn1c* (Bhogal et al. 2004), we concluded that methylation at this region does not participate in tooth development.

## Discussion

The present study demonstrated a role for p57 in murine tooth development at early postnatal stages. Data from the GEO database showed that the expression pattern of p57 changes dynamically during different stages of tooth development (Fig. 1). In our experiment, we suggest that p57 and the methylation status of the *KvDMR1* region might participate in tooth development, and especially odontoblast differentiation, during the early postnatal stages via the cell cycle inhibitory activity of p57 (Figs. 2, 3, 4).

The *Cip*/*Kip* family members are regarded as critical regulators of odontogenesis (Kumamoto et al. 2001), and are prominently implicated in cell cycle regulation as inhibitors of CDKs (Besson et al. 2008). The *Cip*/*Kip* family contains three members: p21, p27 and p57. p21 is mainly expressed in the inner enamel epithelium during late cap and in the initial bell stages (Bloch-Zupan et al. 1998). p21 is also regarded as a marker of the early phases of odontoblast differentiation (Nakatomi et al. 2013), while p27 was reported strongly expressed in highly differentiated odontoblasts in humans (Klinz et al. 2013). p57 was reported to play a key role in mammalian development by regulating cell proliferation and differentiation in a number of different tissues (Pateras et al. 2009). Nevertheless, the functional role of p57 in odontogenesis remains unexplored. The GEO data we analyzed showed that the expression of p57 in developing tooth germ was spatiotemporal and tissue-specific (Fig. 1). This finding indicated that p57 might play a distinct role in tooth development.

There are five stages of tooth development: thickening stage, bud stage, cap stage, bell stage, and secretory stage (Tucker and Sharpe 2004; Zhang et al. 2018). In the Balb/c model, first molars at P1 and P3 were at the late bell stage, and at P5 and P7 were at the secretory stage (Fig. 2a–d), similar to a previous report (Lv et al. 2011; Jiang et al. 2017). The bell stage is a crucial stage where epithelial-mesenchymal interactions culminate in dramatic morphological and functional changes (Liu et al. 2017). During the bell stage, undifferentiated mesenchyme and epithelium differentiate into dentin-secreting odontoblasts and enamel-secreting ameloblasts, respectively (Nakasone et al. 2006). Odontoblasts are a monolayer of cells at the periphery of the dental pulp, and are divided into three types: preodontoblasts, immature odontoblastas, and mature odontoblasts (Quispe-Salcedo et al. 2012). Preodontoblasts are the polarized cells beginning to stop their proliferation and acquire differentiation, while mature odontoblasts are matrix-producing cells with their differention almost completed. It is generally thought that there exists an inverse relationship between cell proliferation and differentiation, and p57 is a cell cycle arrest gene and is associated with exit from the cell cycle and entry into differentiation (Rossi et al. 2018). Thus it is explicable that we observed intensely p57 expression in immature odontoblasts, and reduced expression in mature odontoblasts (Fig. 2), similar to the expression pattern of p21 (Nakatomi et al. 2013). This result indicated that p57 could serve as a potential marker of early odontoblast differentiation. However, we observed almost no positive staining of p57 in ameloblasts at any stage from P1 to P7, except for some undifferentiated inner enamel epithelium cells at P1 (Fig. 2a5), in contrast to the expression pattern of p21 (Bloch-Zupan et al. 1998). This result suggested that p57 may not participate in the regulation of ameloblast differentiation.

p57 regulates cell differentiation primarily by inhibition of the cell cycle (Martinez et al. 1999). For example, p57 participated in cell cycle exit and differentiation of an oligodendrocyte precursor (Dugas et al. 2007). In addition, during human hair follicle matrix differentiation, p57 is expressed in post-mitotic keratinocytes, accompanying down-regulation of cyclin A and B1 (Purba et al. 2017). In our study, we also observed that p57 expression was negatively correlated with pRB, cyclin D1, and CDK2 during the late bell stage (Fig. 3). However, we discovered both p57 and activity of downstream pathways related to cell cycle were relatively low at P5 and P7. This indicated that p57 might play no significant role in regulating tooth development at P5 and P7.

DNA methylation also plays a vital role in tooth development (Wan et al. 2018; Yoshioka et al. 2015). Our data, however, demonstrate that global methylation levels were relatively low in the tooth germ at P1–P7 (Fig. 4a). In concordance with our results, the global methylation levels were also quite low in the deciduous tooth germ in miniature pigs at the bell stage (E50) and secretory stage (E60); however, many differentially methylated genes were identified (Su et al. 2016). *Cdkn1c* lies within a complex imprinted domain, and is regulated by an imprinting center, *KvDMR1*, in both humans and mice. Methylation at this locus inhibits the expression of *Kcnq1ot1* but allows the expression of *Cdkn1c* (Eggermann et al. 2014). The absence or loss of methylation at this locus results in Beckwith-Wiedemann syndrome leading to growth disorders (Bastaki et al. 2016; Singh et al. 2017). In addition, *Cdkn1c* is also regulated by the DMR of *Cdkn1c* in mice, which is located at the promoter and gene body (Bhogal et al. 2004). Methylation at this locus leads to inhibition of *Cdkn1c* expression. In the present study, we discovered that differential methylation of *KvDMR1*, not *Cdkn1c* sDMR, regulates the expression of *Cdkn1c* in tooth development (Fig. 4c). Similar expression patterns were also found in the liver, spleen, and lung of cattle (Wang et al. 2015). Since the methylation levels at the *Cdkn1c* sDMR are not a precondition for its imprinted expression, but likely are essential to maintain its imprinting (Bhogal et al. 2004), we suggest that methylation of the *KvDMR1* region, but not the *Cdkn1c* sDMR region, is involved in tooth development.

In conclusion, the present study sheds light on the role of p57 in the early postnatal stage of tooth development. We report a detailed observation of the spatiotemporal expression of p57 in tooth germ, and we explore the potential molecular mechanisms by which it governs tooth development. We also examine the methylation status of the two DMR regions of *Cdkn1c*, and we demonstrate that *Cdkn1c* plays a crucial role in odontoblast differentiation. However, since the direct molecular mechanisms and functional analysis did not investigate in our studies, the elucidation of a potential role of *Cdkn1c* in tooth development, especially in odontoblasts, requires further more complex studies.
